# Multimodality Imaging Assessment of Ischemic-Related Submitral Left Ventricular Pouch: A Case Report

**DOI:** 10.7759/cureus.38575

**Published:** 2023-05-05

**Authors:** Achraf Machraa, Oumaima Fertat, Walid Ben Brahim, Latifa Oukerraj, Mohamed Cherti

**Affiliations:** 1 Department of Cardiology B, Ibn Sina University Hospital Center, Mohammed V University, Rabat, MAR

**Keywords:** multimodal imaging, ischemic heart disease, aneurysm, left ventricular, submitral

## Abstract

Submitral left ventricular aneurysm remains a rare condition with a varied etiology besides the congenital origin. We describe the case of a 62-year-old male patient who presented, two weeks after an inferobasal myocardial infarction (MI), with dyspnea and atypical chest pain. Transthoracic echocardiography (TTE) and cardiac computed tomography (CT) revealed a giant thin-walled submitral left ventricular aneurysm. He was managed conservatively given the high operative risk. The overall survival was five months after discharge. Despite its rarity, recognizing the causal relationship between ischemic heart disease and submitral aneurysm can prevent life-threatening complications and is therefore of major importance. In the era of advanced imaging, multimodality cardiac imaging techniques are a key element for guiding diagnostic and therapeutic strategies.

## Introduction

Submitral left ventricular aneurysm is an uncommon cardiac condition first described in native African populations [1]. It is mostly considered to be congenital in origin; however, multiple associations with myocardial infarction (MI), traumatic, or diverse inflammatory and infectious diseases, such as tuberculosis and Takayasu's arteritis, were described [2].

Clinical presentations can range from symptoms of heart failure, which are usually due to mitral regurgitation or left ventricular dysfunction, to sudden cardiac death in cases of ventricular wall rupture or ventricular arrhythmias [3].

We report the case of a 62-year-old man with a fast-expanding submitral aneurysm following an inferobasal MI and highlight the importance of a multimodal imaging approach to guide diagnostic and therapeutic strategies.

## Case presentation

**Figure 1:**
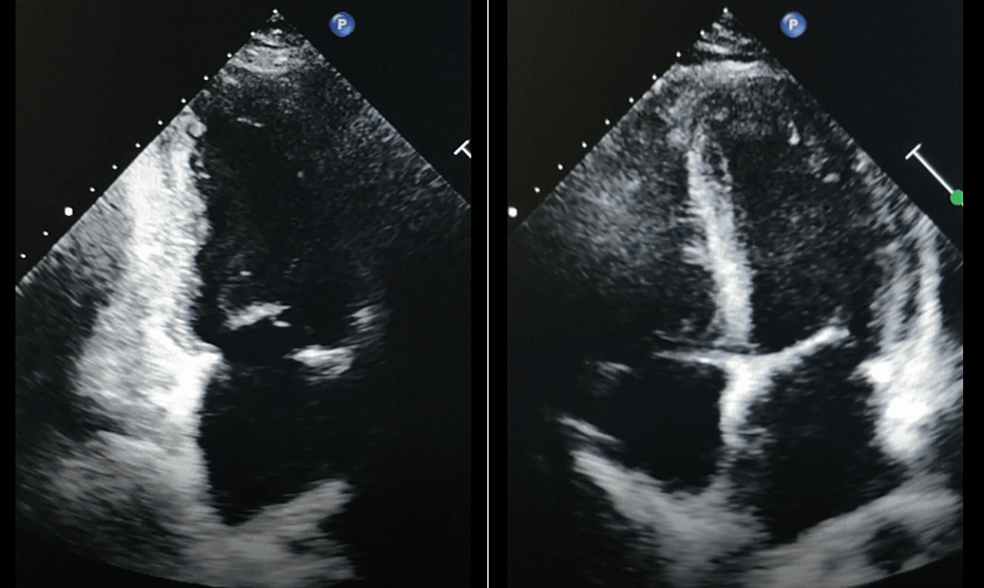
TTE showing the continuity of the myocardial wall without apparent structural abnormality. TTE: transthoracic echocardiography.

A 62-year-old man, an active smoker (30 pack-years), with a medical history of diabetes, presented to his local emergency department 16 hours after the onset of acute and constant chest pain radiating into his left arm. The pain resolved after administration of glyceryl trinitrate spray sublingually. He was then referred to our department because of an electrocardiogram showing ST-segment elevation in inferobasal leads with a troponin peak of 35 ng/mL (N < 0.39 ng/mL). His coronary angiography revealed multivessel coronary artery disease with severe stenosis of the proximal right coronary artery (RCA), followed by thrombotic occlusion of the mid RCA with Rentrop grade 2 collaterals providing retrograde flow to the distal RCA from the left coronary network, and significant stenosis involving the distal left main, the mid and distal left anterior descending (LAD), and the left marginal arteries. A decision was made to refer the patient for a coronary artery bypass graft (CABG) procedure. He was discharged with dual antiplatelets, statins, beta-blockers, and angiotensin-converting enzyme inhibitors (ACEIs). The discharge transthoracic echocardiography (TTE) demonstrated akinesia in the inferior and inferolateral walls, minor mitral regurgitation, and a mildly reduced left ventricular ejection fraction (48%) (Figure 1).

**Figure 2:**
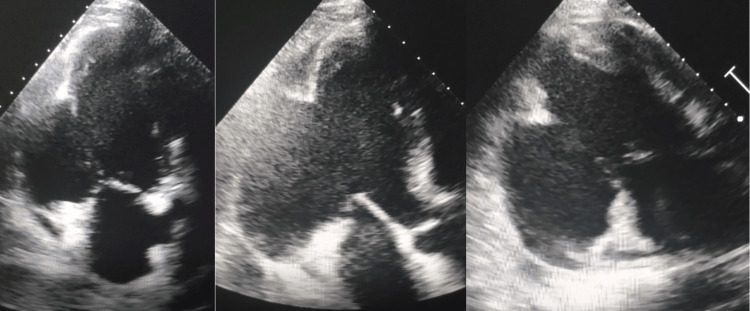
TTE showing a large submitral aneurysm free of thrombus with a wide neck. TTE: transthoracic echocardiography.

He was readmitted seven days after discharge due to a gradually worsening dyspnea on exertion with atypical chest pain. Physical examination showed a heart rate of 75 beats/min, blood pressure of 120/63, bilateral basal crackles, and a third heart sound (S3). TTE disclosed a large submitral aneurysm free of thrombus with a wide neck and mild mitral regurgitation (Figure 2).

**Figure 3:**
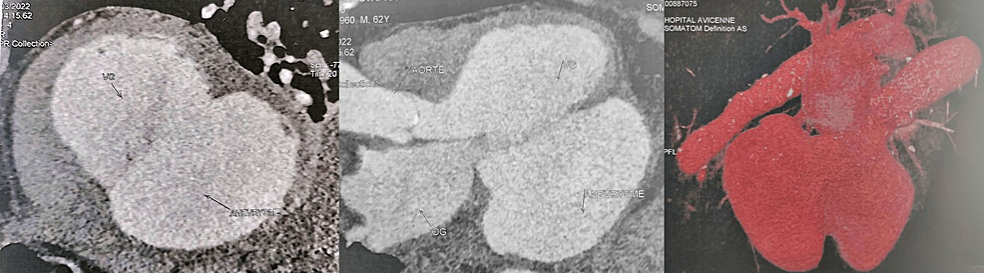
Cardiac CT showing a giant thin-walled (3 mm) submitral aneurysm (97 x 74 mm) free of thrombus with a wide neck (67 mm) and no thrombus or pericardial effusion.. CT: computed tomography.

A cardiac CT confirmed the TTE findings, and with 2D-3D maximum intensity projection (MIP) images, it helped define the exact anatomy, size, and relationship with the surrounding structures. The cardiac CT images revealed a giant thin-walled (3 mm) aneurysm at the basal region near the mitral valve, measuring 97 x 74 mm with a wide neck of 67 mm and no thrombus or pericardial effusion (Figure 3).

A multidisciplinary meeting was conducted to decide upon patient management. Knowing the poor prognosis of an enlarging left ventricular aneurysm, surgical repair was considered by the local heart team but discarded due to the high procedural risks and the high probability of mitral valve repair or replacement because of the proximity to the mitral annulus. Percutaneous closure by an occluder device was not feasible given the anatomy of the aneurysm with a large neck, which is predictive of a lack of adherence of the device along its neck.

In the absence of in-hospital complications, he was discharged with optimal medical therapy (anticoagulants, statins, beta-blockers, ACEIs, aldosterone antagonists, sodium-glucose co-transporter 2 inhibitors) . At follow-up, TTE evaluation showed stability of the aneurysm. His overall survival was five months after discharge. We can only think of the cause of death as either aneurysm rupture or ventricular arrhythmia.

## Discussion

Most of the available evidence about submitral left ventricular aneurysm is from Africa, suggesting that both common genetic and environmental etiologies should also be considered because of racial predilection, with frequent occurrence among African populations [4].

As described by Sharma and Kumar [2], the etiology of left ventricular aneurysm is classified as: congenital aneurysm and acquired aneurysm, which can be classified into ischemic, traumatic, postcardiac surgery, infective, and inflammatory. However, the etiology of submitral aneurysms has always been considered congenital rather than ischemic, as only a few cases could identify coronary artery disease as the cause.

While 85% of left ventricular aneurysms involve the anterior wall, posterior wall aneurysms are very infrequent. Clinical manifestations can range from heart failure to thromboembolic events, ventricular arrhythmias, and ventricular wall rupture [5,6]. In our case, the main complaint was dyspnea. This can be explained by the fact that the left ventricular aneurysm represents more than 20% of the left ventricular circumference, which is predictive of the occurrence of congestive heart failure. The unusual localization of the submitral aneurysm is a source of valvular complications since the growth of a subvalvular aneurysm leads to distortion of the mitral annulus or the subvalvular apparatus, which results in mitral regurgitation.

The greatest diagnostic challenge for clinicians is distinguishing between a true left ventricular aneurysm, which is formed by the full thickness of the ventricular wall, and a pseudo aneurysm, which is defined as a rupture of the ventricular wall contained by the overlying pericardium, scar tissue, and thrombus.

Despite the fact that TTE is the first line and a sufficient diagnostic approach, it provides information on aneurysm size, location, extent, neck size, presence of thrombus, mitral regurgitation severity, and ventricular contraction. Multimodality cardiac imaging, such as CT and magnetic resonance imaging (MRI), can give a better view of the aneurysm wall, help in delineating the length of the neck, identify complications, and help in planning management strategies by assessing the resectability [7].

Management includes stabilization of heart failure with medical therapy and anticoagulation to prevent systemic thromboembolism. Surgical repair includes extra- or intracardiac repair with pericardial patch placement and mitral valve repair or replacement. Surgical outcomes are poor, and the size of the left ventricular residual cavity at the end of the intervention is critical for the patient's outcomes. Percutaneous closure of the left ventricular aneurysm is a feasible alternative for high-risk surgical candidates [8]. In such cases, multimodality imaging is mandatory for a proper assessment of the anatomy, for planning the procedure and selecting the appropriate closure device.

Despite the available guidelines regarding the benefit of complete revascularization in ST-segment elevation MI patients with multivessel disease before discharge. In our case, referring the patient for CABG appeared as the preferable option at the time of the first hospitalization, taking into account our local context.

## Conclusions

Submitral left ventricular aneurysm remains a rare condition with a varied etiology. Our case highlights the causal relationship between ischemic heart disease and submitral aneurysm. Multimodality cardiac imaging techniques are a key element in guiding the therapeutic approach. Surgical repair should always be considered as the primary option since mortality rates are even higher with medical management.
